# Connecting functional and statistical definitions of genotype by genotype interactions in coevolutionary studies

**DOI:** 10.3389/fgene.2014.00077

**Published:** 2014-04-11

**Authors:** Katy D. Heath, Scott L. Nuismer

**Affiliations:** ^1^Department of Plant Biology, University of IllinoisUrbana, IL, USA; ^2^Department of Biological Sciences, University of IdahoMoscow, ID, USA

**Keywords:** coevolution, symbiosis, pathogen, epistasis, intergenomic epistasis

## Abstract

Predicting how species interactions evolve requires that we understand the mechanistic basis of coevolution, and thus the functional genotype-by-genotype interactions (G × G) that drive reciprocal natural selection. Theory on host-parasite coevolution provides testable hypotheses for empiricists, but depends upon models of functional G × G that remain loosely tethered to the molecular details of any particular system. In practice, reciprocal cross-infection studies are often used to partition the variation in infection or fitness in a population that is attributable to G × G (statistical G × G). Here we use simulations to demonstrate that within-population statistical G × G likely tells us little about the existence of coevolution, its strength, or the genetic basis of functional G × G. Combined with studies of multiple populations or points in time, mapping and molecular techniques can bridge the gap between natural variation and mechanistic models of coevolution, while model-based statistics can formally confront coevolutionary models with cross-infection data. Together these approaches provide a robust framework for inferring the infection genetics underlying statistical G × G, helping unravel the genetic basis of coevolution.

## Introduction

Coevolution, or reciprocal evolutionary change between two or more species and has shaped key early branches in the tree of life, including the origin of the eukaryotic cell from endosymbiosis (Sagan, 1967; Doolittle, 2000). Coevolution can drive diversification and promote biodiversity, as exemplified by the diverse floral forms and secondary chemistry among contemporary plants (Sagan, 1967; Doolittle, 2000; Futuyma and Agrawal, 2009; Yoder and Nuismer, 2010). In addition, coevolutionary interactions shape ecosystem-level processes (e.g., the terrestrial nitrogen cycle via the symbiosis between leguminous plants and nitrogen-fixing bacteria; Graham and Vance, 2003), have major economic importance (e.g., crop pollinators and pests; Oerke, 2006), and impact human health (e.g., bacterial antibiotic resistance; Toprak et al., 2011). The importance of these processes to the world around us is well-known; nevertheless, we are only now beginning to develop an adequate framework for understanding and predicting how these interactions evolve and coevolve (Thompson, 2005). As anthropogenic changes such as climate warming, nitrogen deposition, and prevalent antibiotic use alter the context in which species interactions evolve (Six, 2009; Kiers et al., 2010; Northfield and Ives, 2013), society will benefit from an improved understanding of the coevolutionary process. Among other things, a predictive understanding of coevolution would help us manage coevolutionary phenotypes, like antibiotic resistance or biological nitrogen fixation, that are critical to human health and agriculture.

### Functional G × G

**Figure 1:**
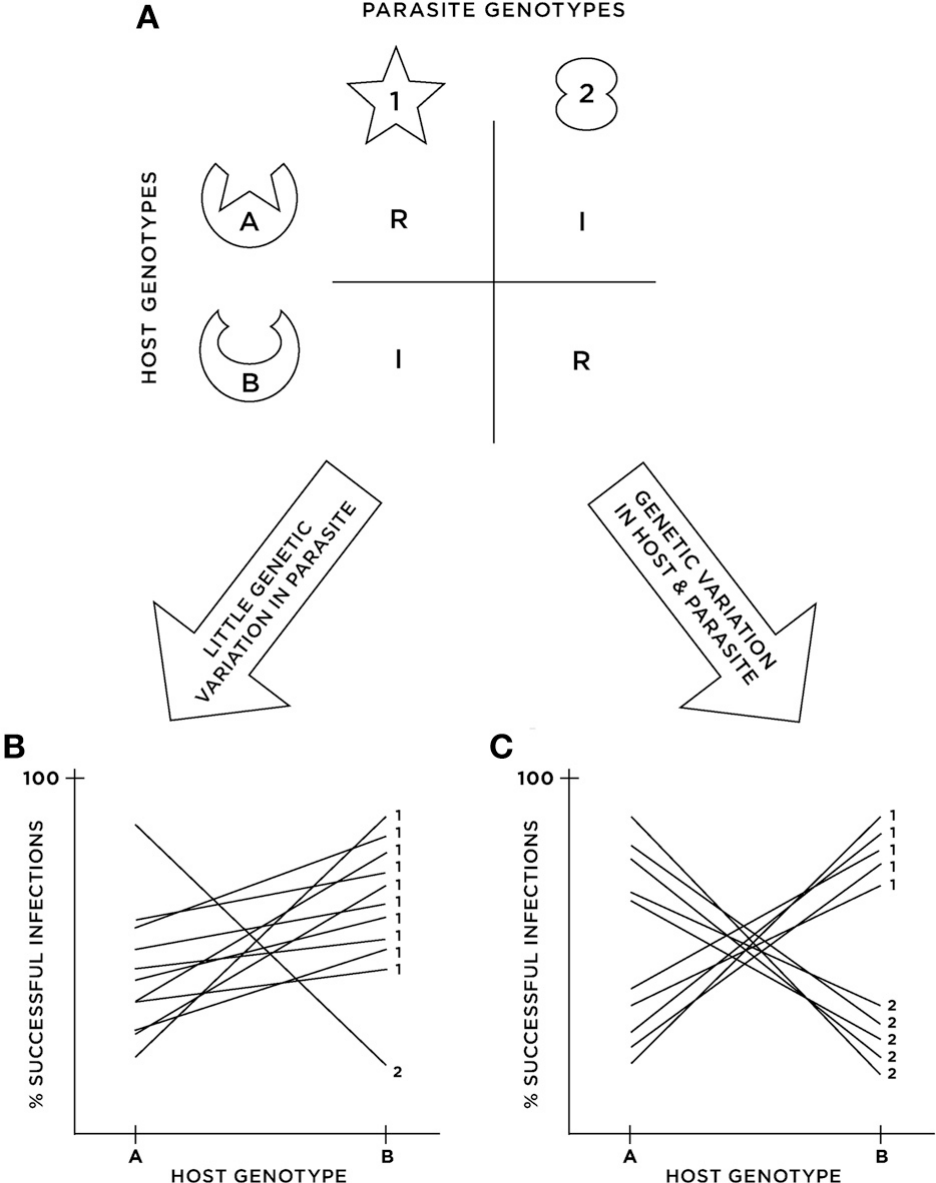
**Cartoon illustrating the potential disconnect between functional and statistical G × G. (A)** Depicts an example of functional G × G where a “match” between host and parasite molecules triggers host defense, resulting in resistance (i.e., inverse matching alleles or IMA). **(B,C)** Depict the results of cross-infection studies in two different populations possessing the functional G × G in **(A)**. In **(B)**, parasite genotype 2 is rare in the population, resulting in a small G × G variance component, and thus an inferred lack of statistical G × G when analyzed with ANOVA. In **(C)**, parasite genotypes are in equal frequencies in the population, resulting in a large G × G variance component and thus strong evidence for statistical G × G.

Because coevolution requires reciprocal evolutionary change between interacting species (Janzen, 1980), evolutionary change in Partner A must beget evolutionary change in Partner B, which in turn must beget evolutionary change in Partner A, and so on and so forth. From a genetic perspective, this means that changes in genotype frequencies in Partner A alter the relationship between genotypes and fitness in Partner B (and vice versa). This can only occur if there are genotype × genotype (G × G) interactions for fitness-related traits in the two species, or what we term “*functional G × G*” following the recent analysis of epistasis by Hansen (2013). Functional G × G can be driven by a direct interaction between gene products of the interacting species (e.g., plant or animal receptors that recognize pathogen molecules; see Figure 1A and review by Ausubel, 2005) or by the interaction between quantitative traits to which the genotypes contribute (e.g., snake resistance and newt toxicity; Brodie and Ridenhour, 2003). The mechanistic underpinnings of such functional G × G, particularly the genetic basis of infection in host-parasite interactions, have long been a subject of debate (e.g., matching alleles vs. gene-for-gene models; Frank, 1996; Parker, 1996; Agrawal and Lively, 2002). These mechanistic details dramatically alter model-based predictions (e.g., Poullain and Nuismer, 2012), which many empiricists seek to address using their own coevolutionary systems. Thus, assessing both the existence and form of functional G × G are important goals toward a predictive understanding of coevolution.

### Statistical G × G

Although, in some cases, it is possible to study the direct, functional interactions between genotypes that fuel coevolution, in many others this is simply not possible. As a consequence, alternative approaches have been developed and applied to a wide range of natural species interactions. One commonly employed approach relies on reciprocal cross-infection experiments in which some number of genotypes (isolates/strains, families, inbred lines, etc.) from both partners are tested against each other in a factorial design, using genotypes sampled from either a single location or from various locations (Ebert et al., 1998; Lambrechts et al., 2005; Salvaudon et al., 2007; Sandrock et al., 2010; Bryner and Rigling, 2011; Lemaire et al., 2012; Cayetano and Vorburger, 2013). The resulting data are typically analyzed within an ANOVA framework to partition the variation in infection (and/or other traits) attributable to host genotype (*G*_*h*_), parasite genotype (*G*_*p*_), and their interaction (*G*_*h*_ × *G*_*p*_). Variation among populations can also be estimated in studies of multiple locations (e.g., Carius et al., 2001; Heath, 2010; Thrall et al., 2012). In practice, therefore, empiricists implicitly apply the variance-partitioning framework of quantitative genetics (Falconer and Mackay, 1996; Lynch and Walsh, 1998) to populations in order to estimate (or simply test the significance of) the G × G variance component, which we term “*statistical G × G*” to emphasize that it is a population-level quantity that depends on genotype frequencies (Figures 1B,C). Consequently, there need be no direct mapping between the mechanistic G × G interactions that are critical for coevolution and the statistical G × G interactions detected using cross-infection studies. Nevertheless, statistical G × G has sometimes been invoked as a necessary prerequisite for coevolution, either implicitly or explicitly (Parker, 1995; Heath, 2010; Sandrock et al., 2010).

### Reconciling functional and statistical G × G

The lack of a direct mapping between functional and statistical interactions raises important questions regarding the interpretation of statistical estimates of G × G derived from reciprocal cross infection studies. For instance: (1) Is statistical G × G always present in coevolving populations? (2) Is significant statistical G × G an indicator of coevolution, or the strength of coevolution? and (3) Do estimates of statistical G × G provide information about the form of functional G × G underlying coevolution? Here we answer these questions using coevolutionary models that allow us to formally connect different types of functional G × G to the patterns of statistical G × G they produce within a single population. Our brief modeling exercise demonstrates that, because of both sampling error and the coevolutionary process itself, these two definitions of G × G are likely to be loosely related at best. We finish by suggesting some future approaches for merging empirical cross-infection data with theoretical models to better understand the mechanistic basis of coevolution.

## Simulations: reciprocal cross infection studies of coevolving populations

One way to evaluate the link between G × G at the individual level and statistical G × G measured at the level of a population is to develop and analyze mathematical models of the coevolutionary process. Our basic approach is to first simulate coevolution using simple, well-studied models of host-parasite interactions. These models assume coevolution is mediated by a single locus in each species, with the outcome of encounters between individuals described by one of several well-studied genetic models (details in Data Sheet 2 in Supplementary Material). As “controls,” we also simulate host-parasite interactions under models that: (1) lack functional G × G and thus the potential for coevolution, and (2) eliminate coevolutionary selection by setting the fitness effects of infection to zero (see details in Data Sheet 2 in Supplementary Material).

After simulating coevolution, we analyze the simulated populations as if we were conducting an empirical reciprocal cross-infection experiment. Specifically, for a subset of simulated generations of the simulation, we sample host and parasite genotypes at random and challenge them with one another using a within-population reciprocal cross-infection design. We then partition the variance in the outcome of these interactions (infect vs. resist) into components corresponding to error, host genotype, parasite genotype, and the interaction between host and parasite genotype (i.e., statistical G × G). A much more detailed description of coevolutionary simulations and simulated cross-infection experiments can be found in Data Sheet 2 in Supplementary Material.

### Is statistical G × G always present in coevolving populations?

**Figure 2:**
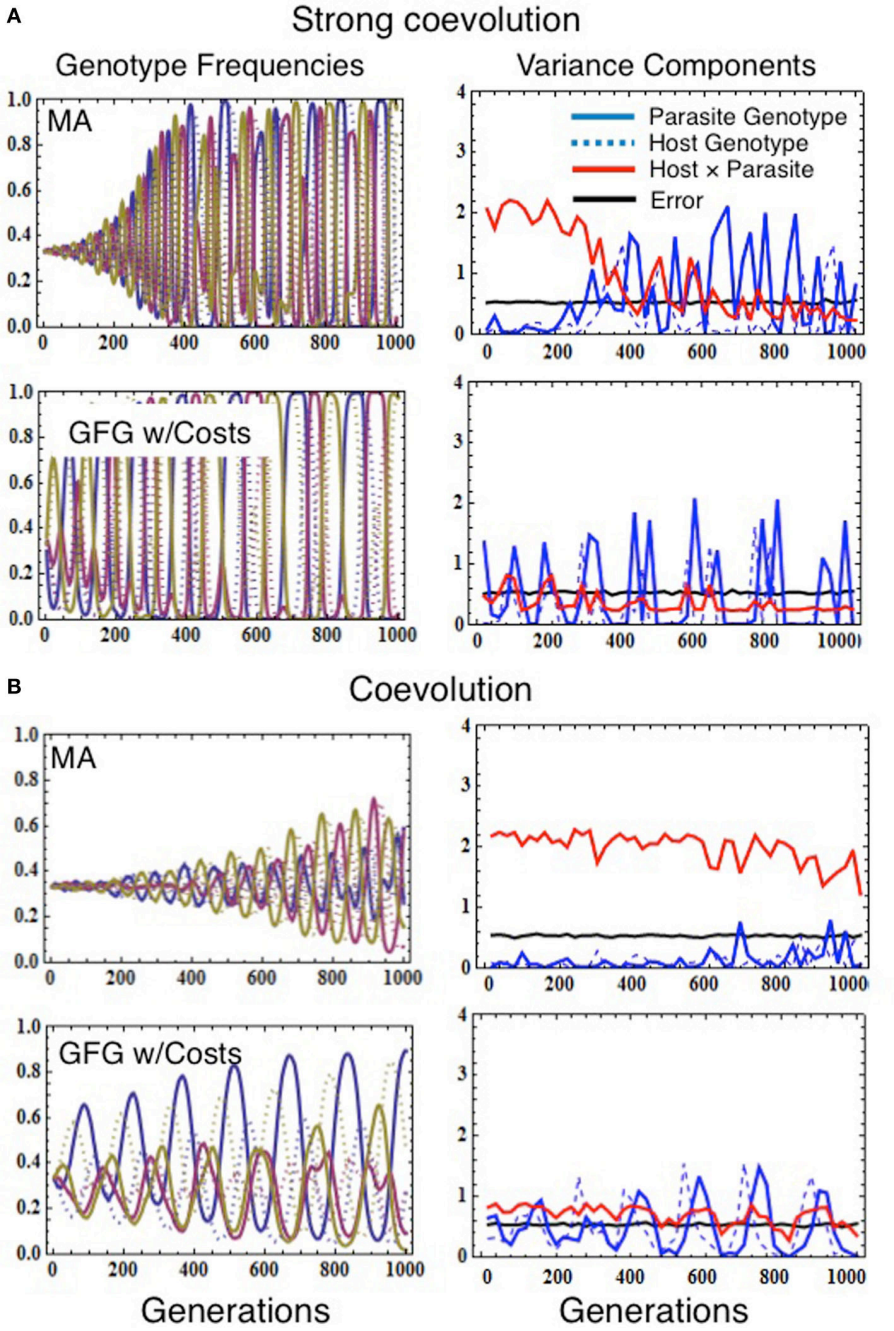
**Simulations demonstrating how the various models of infection genetics (matching alleles vs. gene-for-gene) affect population allele frequencies (left column) and variance components estimated from simulated cross-infection studies (right column), in the presence of coevolution**. Evolutionary simulations assumed the population size of both host and parasite was 100,000, the mutation rate for both species was 1 × 10^−5^, the number of genotypes in both host and parasite was 3, and that the fitness consequences of interactions were set to *s*_*h*_ = 0.67 and *s*_*p*_ = 0.69 (for strong coevolution, **A**) or *s*_*h*_ = 0.37 and *s*_*p*_ = 0.39 (coevolution, **B**). Costs of resistance and virulence in the GFG model were set to τ_*h*_ = 0.12 τ_*p*_ = 0.09 **(A)** or τ_*h*_ = 0.08 τ_*p*_ = 0.05 **(B)**. Simulated reciprocal cross-infection studies were performed every 20 generations by sampling 30 host and parasite genotypes. Each genotype was then used to calculate the number of infections occurring in five trial exposures. This experiment was replicated five times for each combination of host and parasite genotypes. Variance components were then estimated as described in Data Sheet 2 in Supplementary Material.

Absolutely not. We found that low/negligible statistical G × G is quite common even when coevolution is a very strong force shaping allele frequencies in a population (Figure 2A). This is because, during strong coevolutionary cycles, host and parasite genotypes cycle close to fixation before negative frequency-dependent selection decreases their frequencies once again (Seger, 1988; Frank, 1992). Allele frequency cycles inhibit detection of statistical G × G for two reasons. First, as alleles become rare, they are less likely to be sampled in cross-infection studies (magnifying the effects of population sampling error; Frank, 1996). The second driver is coevolution itself, since quantitative genetic variance components depend on the standing genetic variation in the population and thus on the evenness of the genotype frequencies (Falconer and Mackay, 1996). Thus, when coevolution leads to large amplitude cycles in genotype frequency (Figure 2A), genetic variation fluctuates over time causing concomitant fluctuations in statistical G × G. Both sampling error and the cyclical nature of host-parasite coevolution, therefore, can cause statistical G × G to be negligible at many points in time even when coevolutionary selection is actually the major force driving evolution within the interacting populations over relatively long time scales. Similar results would be obtained for coevolutionary models characterized by repeated selective sweeps of novel advantageous alleles generated by sustained escalatory coevolution (Sasaki, 2000; Agrawal and Lively, 2002; Brodie et al., 2002; Hall et al., 2011). Although our results demonstrate that an absence of statistical G × G at any single point in time does not indicate an absence of coevolution, they do provide compelling evidence that, if significant statistical G × G is detected, functional G × G must exist (σ^2^_error_ is always much larger than σ^2^_host_ × parasite in Supplementary Figure 1).

### Is significant statistical G × G an indicator of coevolution, or the strength of coevolution?

Unfortunately, no. As discussed above, strong coevolutionary cycles themselves contribute to a disconnect between the coevolutionary selection acting on host and parasite alleles and the population-level statistical G × G. This generates a seemingly-paradoxical result: the stronger coevolutionary selection, the greater the amplitude of genotype frequency cycles, and the more frequent are periods of time in which statistical G × G is negligible (i.e., compare Figure 2A to Figure 2B, in which coevolutionary cycles are weaker due to decreased strength of selection on both hosts and parasites). There is, therefore, no direct mapping between the strength of statistical G × G interactions inferred from reciprocal cross-infection studies conducted within single populations and the intensity of coevolution at multi-generation time scales. Indeed even when coevolution is weak or absent because the selective impacts of infection/resistance are minimal or non-existent, it is quite possible to detect very strong statistical G × G interactions in traits of interest (Supplementary Figure 2). This result emphasizes the importance of connecting traits to their fitness effects for both partners in a coevolutionary interaction.

### Do estimates of statistical G × G provide information about the form of functional G × G underlying coevolution?

Yes, but not much. The significance or magnitude of statistical G × G reveals little about the underlying form of functional interactions between host and parasite genotypes; instead, statistical G × G simply indicates that functional G × G exists (at least for some genotypes). When large amplitude cycles are produced by coevolution (i.e., later generations in Figure 2), different functional models of G × G converge on similar variance components. Although differences in the magnitude or frequency (across generations) of statistical G × G do exist among models (e.g., GFG generally generates less statistical G × G on average), all generate substantial statistical G × G in at least some situations. Given that the typical empiricist samples a population at only one or a few time points, it would be virtually impossible to achieve the multi (here, 1000)-generation view necessary to distinguish among different functional models of G × G.

### Implications of simulation results

We explored the connection (or, rather, lack thereof) between the functional models of G × G that drive coevolution, the strength of coevolution, and the population-level (statistical) G × G often estimated by empiricists. While statistical G × G is indicative of functional G × G, our results suggest an absence of statistical G × G should rarely, if ever, be used to suggest coevolution is not occurring. Thus, while previous studies of statistical G × G (see references above) are not inherently flawed, our results would suggest a strong false-negative bias, since *not* finding statistical G × G might be common even in coevolutionary systems. In fact, our simulations suggest that the strongest coevolutionary cycles in nature might be the least likely to result in within-population statistical G × G in cross-infection studies.

Although we have not performed the relevant simulations, we suggest that these conclusions are also likely apply to the selection mosaic, defined as G × G × E (Thompson, 2005; Gomulkiewicz et al., 2007). Because, like G × G, the ability to detect G × G × E also rests upon genotype frequencies in the contemporary population at sampling time, and upon the probability of sampling those genotypes from nature, not finding it in any particular generation or experiment is unlikely to be a very useful indicator that selection mosaics do not exist. Finally, our simulations suggest that, in general, it will not be possible to differentiate between functional models of G × G (e.g., GFG, MA) using only statistical estimates of G × G drawn from reciprocal cross-infection studies conducted within single populations. Other approaches will be necessary (see below).

### Limitations of our approach

Our results show that because statistical G × G depends on population genotype frequencies, coevolutionary dynamics themselves can decouple statistical and functional G × G. We specifically used parameter values tuned to dynamically maintain genetic variation (e.g., rapidly-oscillating genotype frequencies in Figure 2). While not uncommon in the theoretical literature (Seger, 1988; Lively, 1999), very strongly oscillating dynamics such as those modeled here might be rare in nature if coevolutionary selection is generally weak, or if coevolution tends to be driven more by escalation rather than negative frequency dependence. Other factors, such as gene flow among populations or more complex genetic architectures of defense and counter-defense might also reduce the likelihood of high amplitude oscillations. However, at least in some cases, empirical data do support the basic prediction of fluctuating genotype frequencies resulting from negative frequency-dependent selection (Koskella and Lively, 2009; Tack et al., 2012; Thrall et al., 2012). In addition, it seems quite likely that our general predictions would hold for any form of coevolution that causes the genotypes mediating the interaction to fluctuate in frequency over time.

## Where do we go from here?

Together, the results of our simulated experiments suggest limitations on what we can hope to learn from statistical estimates of within-population G × G drawn from reciprocal cross infection studies. These results emphasize the importance of integrating multiple populations into reciprocal cross-infection studies (reviewed by Greischar and Koskella, 2007; Hoeksema and Forde, 2008; Nuismer and Gandon, 2008) or multiple points in time as has been done in some unique longitudinal cross-infection studies (Decaestecker et al., 2007; Thrall et al., 2012; Blanquart and Gandon, 2013) in order to test key predictions of coevolutionary theory. Nevertheless, interpreting the existence (or lack thereof) of within-population statistical G × G in these more powerful cross-infection designs is likely to suffer from problems similar to those we identified for single population/time point studies. For this reason, we now outline some promising approaches for wringing more information about the coevolutionary process from reciprocal cross-infection studies.

### Model-based statistical inference

One way to glean more valuable information from reciprocal cross-infection studies is to move toward model-based statistical analyses rather than traditional analyses based on variance partitioning. Perhaps the simplest way such model-based approaches could be implemented is by directly calculating the likelihood of the reciprocal cross-infection data given a particular functional model of G × G interactions (i.e., GFG, MA; Data Sheet 2 in Supplementary Material). Likelihood ratio tests could then be used to evaluate the relative support for the various candidate models. A strength of this approach is that the number of segregating genotypes within each species, and the frequency of these genotypes, is simultaneously estimated during the likelihood calculations using nothing but the cross-infection data. Although computationally straightforward for small numbers of genotypes, the likelihood calculations become demanding very quickly as the number of possible genotypes increases. When practicable, however, such an approach would yield much more information about the form of functional G × G from cross-infection datasets, compared to conventional variance decomposition. Most notably, this approach could be used to directly support/reject differing models of functional G × G using the empirical data typically used to estimate statistical G × G (MA, GFG, etc.).

### Identification of underlying genetic basis

Finding statistical G × G for fitness or fitness-associated traits in cross-infection studies is also a first step toward unraveling the functional basis of coevolutionary mechanisms, i.e., by screening for particular individual genotypes that respond very differently from others and thus identifying the lines/strains possessing the critical functional G × G. Phenotyping the outcomes (e.g., strength or probability of infection) of cross-infection experiments between such genotypes is necessary for mapping studies that seek to find candidate genes or genomic regions responsible for G × G (Wilfert and Schmid-Hempel, 2008; Yang et al., 2008; Gorton et al., 2012; Fansiri et al., 2013). These can then be followed by functional work using molecular techniques such as transformation, overexpression, or RNAi silencing that can validate the role of particular genes or gene variants in determining infection (e.g., Mackey et al., 2003; Yang et al., 2010; Li et al., 2013). Despite many decades of work on host-parasite interactions, we are only beginning to resolve the naturally-segregating variants that actually coevolve in natural populations (reviewed by Barrett et al., 2009; Lambrechts, 2010; Dybdahl et al., in press). Even without molecular biology, when traits are controlled by few genes, segregation ratios estimated in classical genetics studies can be used to test key hypotheses about the infection matrix, such as number of loci and allelic interactions (Little et al., 2006; Luijckx et al., 2013). Leveraging the statistical G × G that exists in natural populations will help us resolve these mechanistic underpinnings, particularly for coevolving traits that are quantitative in nature and thus controlled by many genes of relatively small effect.

### Conflict of interest statement

The authors declare that the research was conducted in the absence of any commercial or financial relationships that could be construed as a potential conflict of interest.
