# Supplementary material for: Connecting functional and statistical definitions of genotype by genotype interactions in coevolutionary studies
Source: Front Genet. 2014 Apr 11;5:77. doi: 10.3389/fgene.2014.00077 (PMC3990044; doi:10.3389/fgene.2014.00077)

Supplementary Figure 1:
No potential for coevolution (host main effects only)

Genotype Frequencies

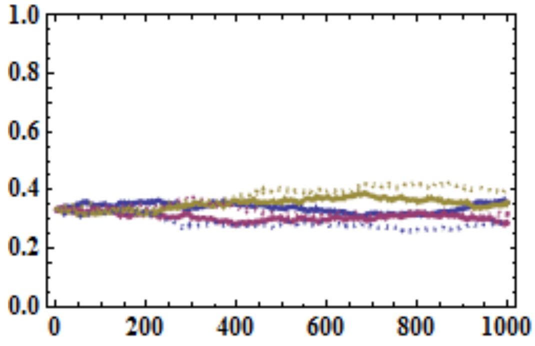

Generations

Variance Components

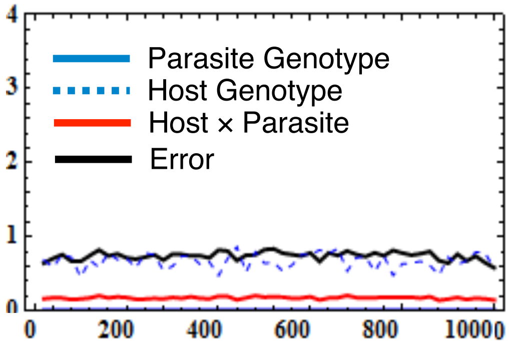

Generations

Supplementary Figure 2: No selection

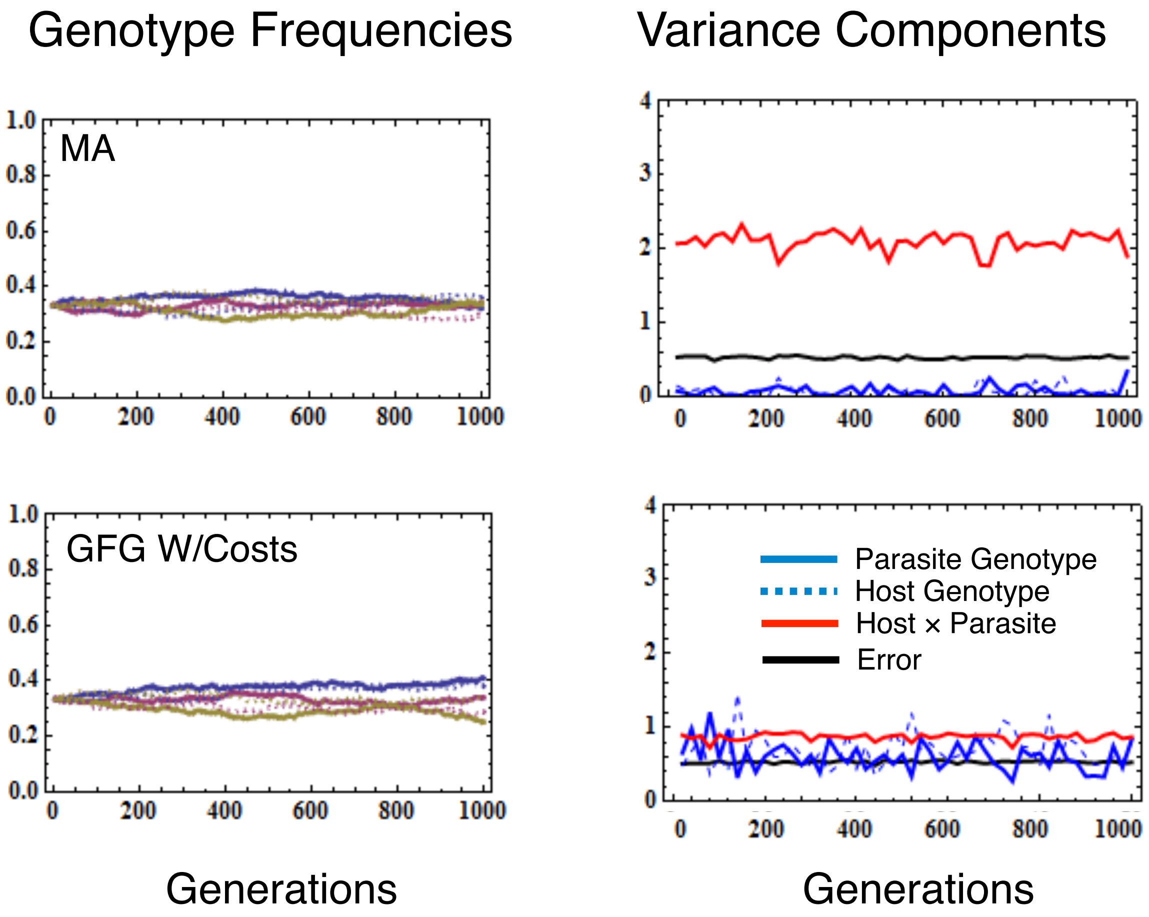

Supplement: Supplementary file 1 [file DataSheet1.PDF]
