# Supplementary material for: Connecting functional and statistical definitions of genotype by genotype interactions in coevolutionary studies
Source: Front Genet. 2014 Apr 11;5:77. doi: 10.3389/fgene.2014.00077 (PMC3990044; doi:10.3389/fgene.2014.00077)
Supplement: Supplementary file 2 [file DataSheet2.DOCX]

**Appendix 1. Description of Coevolutionary Model and Simulated Cross-Infection Experiments**

Coevolutionary model

We study coevolution between a haploid host and parasite. Within each generation, host and parasite individuals encounter one another at random, and the outcome of encounters (infect or resist) depends on the individuals’ genotypes at a single multi-allelic haploid locus. Specifically, we assume that the probability of infection is given by the matrix, **α**, which has host genotypes in rows and parasite genotypes in columns. We will study coevolution when **α** takes the form of three commonly used models of infection and resistance (Supplementary Table 1). When an encounter results in infection, the host individual suffers a fitness reduction, *s_h_* and the parasite enjoys a fitness increase *s_p_*. Together, these assumptions lead to the following expressions for the expected fitness of host and parasite genotypes:

$\mathbf{W}_{\mathbf{h}}\mathbf{=}1-s_{h}\boldsymbol{\alpha P}$ (1a)

$\mathbf{W}_{\mathbf{p}}\mathbf{=}1\mathbf{+}s_{p}\boldsymbol{\alpha H}$ (1b)

where **P** and **H** are vectors describing the frequencies of parasite and host genotypes within the population.

After encounters between the interacting host and parasite populations occur, selective mortality ensues, changing the genotype frequencies within host and parasite populations. Specifically, the frequency of host and parasite genotypes after selection is given by:

$\text{H}^{'}\text{= H}\frac{\text{W}_{\text{h}}}{\bar{W}_{h}}$ (2a)

$\text{P}^{'}=\text{P}\frac{\text{W}_{\text{p}}}{\bar{W}_{p}}$ (2b)

where $\bar{W}_{h}=\text{H.}\text{W}_{\text{h}}$ and $\bar{W}_{p}=\text{P.}\text{W}_{\text{p}}$ are the population mean fitness of host and parasite populations respectively. After selection, mutation occurs at rates *μ_h_* and *μ_p_* in host and parasite, respectively. Mutation is assumed to convert any genotype into any other with equal probability. Finally, randomly selected individuals of each species reproduce until an offspring population of size *N_h_* and *N_p_* is produced. It is at this stage of the life-cycle where random genetic drift occurs. After reproduction, the parental generation dies, and the lifecycle is repeated for *G* generations.

Simulated cross-infection and estimation of variance components

We simulate a reciprocal cross-infection study by sampling from the host and parasite populations modeled by our evolutionary simulations. Specifically, within each generation, we sample ψ host and parasite genotypes at random. Thus, as with many reciprocal cross-infection studies, some genotypes may not be sampled whereas others may be sampled multiple times, introducing realistic population genetic sampling error. After sampling host and parasite genotypes at random, we ran a simulated infection experiment. Specifically, for each host genotype × parasite genotype combination, we ran τ trial exposures and recorded the number of successful infections, S, that occurred. Whether a trial exposure resulted in infection or resistance was determined from the infection matrix, **α**, by drawing a random number (between 0-1) and asking whether it was greater than (resist), or less than (infect), the relevant infection probability in the infection matrix (Supplementary Table 1). By modeling infection/resistance in this way (*i.e.,* rather than as binary 0/1 (match/mismatch) states), we generate experimental error around the mean infection probability for a given host-parasite genotype combination. The infection matrix used in this step was identical to that which governed infection and resistance in our evolutionary simulations (Supplementary Table 1). This procedure was repeated ρ times for each host strain × parasite strain combination, resulting in data of the form shown in Supplementary Table 2. To summarize, we introduce error in two places in our simulated cross-infection study. First, populations of hosts and parasites are sampled randomly, generating an empirically-relevant disconnect between the actual allele frequencies in the population and the genotypes in the cross-infection study in each generation. Second, we introduce random variation around the true probability of infection for each host-parasite genotype combination, generating realistic experimental error.

We used the simulated cross-infection data described in the previous paragraph to partition the variance in the number of successful infections, S, into the following components: host genotype, parasite genotype, host genotype × parasite genotype, and error. This variance partitioning was accomplished using the following standard formulae:

$\mu=\frac{1}{\rho\psi\psi}\sum_{i=1}^{\psi} \sum_{j=1}^{\psi} \sum_{k=1}^{\rho} S_{i,j,k}$ (3a)

$\mu_{i,j}=\frac{1}{\rho}\sum_{k=1}^{\rho} S_{i,j,k}$ (3b)

$\mu_{h,i}=\frac{1}{\rho\psi}\sum_{j=1}^{\psi} \sum_{k=1}^{\rho} S_{i,j,k}$ (3c)

$\mu_{p,j}=\frac{1}{\rho\psi}\sum_{i=1}^{\psi} \sum_{k=1}^{\rho} S_{i,j,k}$ (3d)

$\sigma_{error}^{2}=\frac{1}{\rho\psi\psi}\sum_{i=1}^{\psi} \sum_{j=1}^{\psi} {\sum_{k=1}^{\rho} \left( S_{i,j,k}-\mu_{i,j} \right)}^{2}$ (3e)

$\sigma_{host}^{2}=\frac{1}{\psi}\sum_{i=1}^{\psi} \left( \mu_{h,i}-\mu\right)^{2}$ (3f)

$\sigma_{parasite}^{2}=\frac{1}{\psi}\sum_{i=j}^{\psi} \left( \mu_{p,j}-\mu\right)^{2}$ (3g)

$\sigma_{host\times parasite}^{2}=\frac{1}{\psi\psi}\sum_{i=1}^{\psi} \sum_{j=1}^{\psi} \left( \mu_{i,j}-\left( \mu_{h,i}+\mu_{p,j}-\mu\right) \right)^{2}$ (3h)

where $\mu$ is the grand mean, $\mu_{i,j}$ is the host-parasite genotype combination (cell in **α** matrix) mean, $\mu_{h,i}$ is the host mean, and $\mu_{p,j}$ is the parasite mean.

**Supplementary Table 1.** Genetic models of infection used in simulations. Each cell represents the probability of infection resulting from the genetic interactions between host and parasite alleles (genotypes), under three common genetic models of infection (matching alleles, gene for gene) plus a “control”, in which host and parasite allele effects are additive (*i.e.,* no functional G × G).

| A. Matching alleles | | Host genotype | | |
| --- | --- | --- | --- | --- |
|  |  | 1 | 2 | 3 |
| Parasite genotype | 1 | α_1,1_ = 0.8 | α_2,1_ = 0.2 | α_3,1_ = 0.2 |
|  | 2 | α_1,2_ = 0.2 | α_2,2_ = 0.8 | α_3,2_ = 0.2 |
|  | 3 | α_1,3_ = 0.2 | α_2,3_ = 0.2 | α_3,3_ = 0.8 |

| B. Gene-for-gene | | Host genotype | | |
| --- | --- | --- | --- | --- |
|  |  | 1 | 2 | 3 |
| \|  \| \|  \| \| \| \| --- \| --- \| --- \| --- \| --- \| \|  \|  \|  \| \|  \|  \|  \|  \|  \| \|  \|  \|  \|  \| \|  \|  \|  \|  \|   Parasite genotype | 1 | α_1,1_ = 0.8 | α_2,1_ = 0.2 | α_3,1_ = 0.2 |
|  | 2 | α_1,2_ = 0.8 | α_2,2_ = 0.8 | α_3,2_ = 0.2 |
|  | 3 | α_1,3_ = 0.8 | α_2,3_ = 0.8 | α_3,3_ = 0.8 |

| C. Control | | Host genotype | | |
| --- | --- | --- | --- | --- |
|  |  | 1 | 2 | 3 |
| Parasite  genotype | 1 | α_1,1_ = 0.1 | α_2,1_ = 0.3 | α_3,1_ = 0.5 |
|  | 2 | α_1,2_ = 0.1 | α_2,2_ = 0.3 | α_3,2_ = 0.5 |
|  | 3 | α_1,3_ = 0.1 | α_2,3_ = 0.3 | α_3,3_ = 0.5 |

**Supplementary Table 2.** The structure of data drawn from simulated cross-infection experiments. S_i,j,k_ is the number of infections observed (out of τ exposures) when host genotype i is challenged with parasite genotype j in replicate k. The parameter *ρ* is the number of replicates.

|  | | Host strain/genotype | |
| --- | --- | --- | --- |
|  |  | 1 | 2 |
| Parasite strain/genotype | 1 | S_1,1,1_ = 7  S_1,1,2_ = 8  S_1,1,3_ = 6  $\vdots$  S_1,1,ρ_ = 9 | S_2,1,1_ = 3  S_2,1,2_ = 2  S_2,1,3_ = 1  $\vdots$  S_2,1,ρ_ = 4 |
|  | 2 | S_1,2,1_ = 1  S_1,2,2_ = 1  S_1,2,3_ = 2  $\vdots$  S_1,2,ρ_ = 3 | S_2,2,1_ = 9  S_2,2,2_ = 8  S_2,2,3_ = 9  $\vdots$  S_2,2,ρ_ = 7 |
